# Thoracic epidural analgesia in a patient with von Hippel‐Lindau disease

**DOI:** 10.1002/ccr3.5629

**Published:** 2022-03-22

**Authors:** Amanda Yap, Satoshi Hanada, Sapna Ravindranath, Tejinder Singh Swaran Singh, Yatish Siddapura Ranganath

**Affiliations:** ^1^ 23024 Department of Anesthesiology Eastern Maine Medical Center Bangor Maine USA; ^2^ Department of Anesthesia University of Iowa Carver College of Medicine Iowa City Iowa USA; ^3^ 12250 Department of Anesthesia Indiana University School of Medicine Indianapolis Indiana USA

**Keywords:** anesthesia, epidural, spinal hemangioblastoma, von Hippel‐Lindau disease

## Abstract

von Hippel‐Lindau disease (VHLD) is an autosomal dominant disorder characterized by central nervous system hemangioblastomas and renal tumors. Here, we report a case of thoracic epidural placement in a 35‐year‐old woman with VHLD presenting for left open heminephrectomy for renal masses. We also reviewed the literature on this topic.

## Graphical abstract

Spinal hemangioblastomas in patients with VHLD may be ruptured by neuraxial instrumentation. However, in the absence of spinal hemangioblastoma close to the site of needle entry, neuraxial anesthesia can be used safely. The decision should be made on a case‐by‐case basis with the risks and benefits in mind.

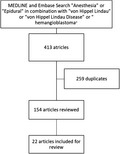

## 1 INTRODUCTION

von Hippel‐Lindau disease (VHLD) has a reported incidence of 1:36,000 live births.^1^ Although it is an uncommon condition, it is not unusual for an anesthesiologist to encounter these patients because they often have multiple surgeries or may be seen in the peripartum period. The hallmark features of VHLD are renal cysts and carcinomas, pheochromocytoma, and hemangioblastomas in the central nervous system and retina.

We report a case of a thoracic epidural placement in a young woman with VHLD who presented for heminephrectomy for a renal tumor. We also conducted a literature review to identify the considerations and practices pertaining to neuraxial anesthesia techniques in these patients.

## 2 CASE PRESENTATION

A 35‐year‐old woman presented for left open heminephrectomy for enlarging renal masses suspicious for carcinoma. The patient's medical and surgical history included VHLD, panhypopituitarism after craniotomy for suprasellar hemangioblastoma resection, left open partial nephrectomy with epidural analgesia, right laparoscopic nephrectomy, left temporal craniotomies for seizures, and suboccipital craniotomy for fourth ventricular hemangioblastoma resection. She had no spine imaging studies performed prior to placement of her last epidural catheter. She had no preoperative neurological or metabolic symptoms. Her medications included acetaminophen, desmopressin, dexamethasone, hydrocortisone, lamotrigine, levothyroxine, lisinopril, and ethinyl estradiol norethindrone. Her most recent electrolytes and complete blood count results were normal with a platelet count of 260,000 mm^3^. Recent abdominal magnetic resonance imaging (MRI) and computed tomography (CT) scans did not demonstrate large spinal hemangioblastomas but were inadequate to detect smaller spinal hemangioblastomas. No formal spine MRI was previously or recently performed. For this surgery, an epidural was requested for postoperative analgesia by the surgical team, which is a common practice for major abdominal surgeries at our institute.

Our primary analgesic plan was for a paravertebral catheter with alternatives, including a transversus abdominis plane or quadratus lumborum blocks. These options, including epidural analgesia, were discussed with the patient. We did not favor epidural catheter placement because of a possible increased risk of spinal hematoma with the potential presence of spinal hemangioblastomas. The patient expressed a strong preference for thoracic epidural placement despite the risks because she had a perception of low pain tolerance and had had an uncomplicated epidural placement in the past. An epidural catheter was placed after considering her clinical history and our knowledge of the temporospatial nature of these hemangioblastomas. We were especially careful to avoid a dural puncture. Using anatomical landmarks, a 20G epidural catheter was sterilely placed at T7‐8 via an 18G, 90 mm Hustead needle in one attempt, and without complications.

Her intraoperative course was uncomplicated. Intraoperatively, a 0.05% bupivacaine infusion at 8 ml/h was started one hour prior to extubation. Postoperatively, she was given patient‐controlled analgesia of hydromorphone and the epidural infusion was increased to 10 ml/h. She was closely monitored with neurological assessment of her lower extremities every two hours for the first day after placement. We increased her epidural infusion rate to 14 ml/h on postoperative day (POD) 1, and eventually removed the epidural catheter on POD 3. She had no focal neurological deficits immediately after the epidural placement, on daily assessment with the epidural in place, at epidural removal, and up to 2 weeks postoperatively.

## 3 DISCUSSION

The primary concern with neuraxial anesthesia in patients with VHLD is the potential risk of rupturing a spinal hemangioblastoma, which is a common feature of VHLD. Accordingly, we considered other alternatives to epidural analgesia such as a transversus abdominis plane (TAP), quadratus lumborum (QL), and paravertebral catheter placement. However, TAP^2^ and QL catheters would require frequent boluses to achieve an adequate level of analgesia and, in this patient, would have been within the surgical field. Thus, these options were not viable. Because spinal hemangioblastomas can also occur at dorsal nerve roots in 0.3% of cases with VHLD, performing a paravertebral block does not eliminate the risk of hemangioblastoma puncture.^3^, ^4^ A prospective, randomized control trial by Schreiber et al. in patients undergoing liver surgery suggests that epidural analgesia provides a modest but significant improvement in pain control compared to paravertebral block catheters.^5^ Therefore, an epidural was likely to be the most effective technique for postoperative analgesia in the presented case. However, the risk accompanied with epidural was difficult to estimate in the setting of no pre‐procedural spine imaging.

An observational histopathological study suggested that in the transverse plane 60% of hemangioblastomas are intermedullary, 11% are intramedullary and extramedullary, 21% are intradural and extramedullary, and only 8% are extradural in location.^6^ In a radiological observational study, 88% of 24 intermedullary tumors were located in the posterior aspect of the spinal cord.^3^ Thus, considering the distribution, the risk might be small with an epidural catheter placement with the needle outside the dura mater. However, we also need to take into account the risk of dural puncture during epidural technique, and the incidence of unintended dural puncture has been reported to be 0.19%–3.6%.^7^ In a prospective observational study of 1278 VHLD‐associated craniospinal hemangioblastomas, 51% remained stable in size whereas 49% exhibited growth, and male sex was also found to be associated with a larger tumor burden and growth.^4^ Based on: (1) the less distribution of spinal hemangioblastomas located in the extradural space^6^; (2) the association of smaller hemangioblastomas being asymptomatic^3^; and (3) our patient's gender, we perceived that the risk of epidural catheter placement in our patient would be acceptably low even without further imaging studies.

**FIGURE 1:**
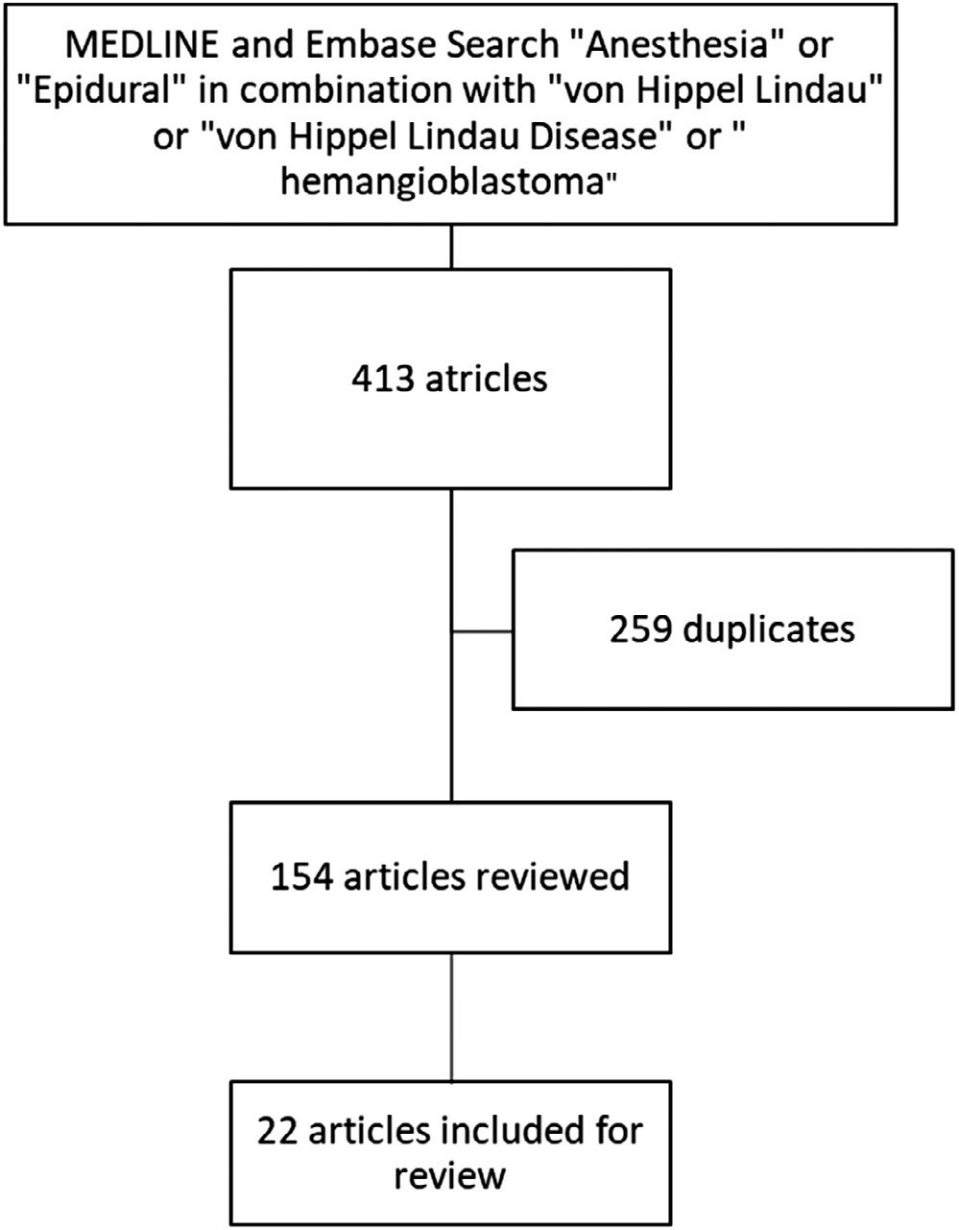
Method of Literature Search

Despite an uncomplicated epidural placement in our patient, we remained inquisitive as to the information that might exist in the literature pertaining to neuraxial anesthesia techniques in patients in VHLD. We therefore performed a literature search using the EMBASE and MEDLINE databases for case reports or series in the English language whereby neuraxial anesthesia techniques were used or discussed in patients with VHLD. We used a combination of the keywords “anesthesia” or “epidural” in combination with “von Hippel Lindau,” “von Hippel Lindau disease,” or “hemangioblastoma.” Our search yielded 413 articles of which 259 were duplicates. The abstracts or texts of the remaining 154 articles were reviewed and 22 articles were included for this literature review (Figure 1) to answer the following questions: (1) Did practitioners obtain pre‐procedural neuraxial imaging study(ies)? (2) Based on their experience, what recommendations had been made regarding performing neuraxial anesthesia techniques in patients with VHLD? (3) What were the outcomes in patients with VHLD who received neuraxial anesthesia techniques?

Of the 22 cases involving VHLD patients, 3 were pediatric patients^8^, ^9^, ^10^ and 19 were adults.^1^, ^11^, ^12^, ^13^, ^14^, ^15^, ^16^, ^17^, ^18^, ^19^, ^20^, ^21^, ^22^, ^23^, ^24^, ^25^, ^26^, ^27^, ^28^ There were a total of 32 surgical procedures performed among the 22 patients, including 2 cases of spontaneous labor. 13 were obstetrical cases,^1^, ^11^, ^12^, ^13^, ^14^, ^16^, ^17^, ^18^, ^20^, ^22^, ^23^, ^25^, ^27^ 10 were neurosurgical procedures,^9^, ^13^, ^14^, ^15^, ^17^, ^19^, ^21^, ^24^, ^25^, ^28^ and 9 were pheochromocytoma resections.^8^, ^9^, ^10^, ^11^, ^15^, ^19^, ^24^, ^26^ 13 of the 22 cases reported single surgical procedures performed^1^, ^8^, ^10^, ^12^, ^16^, ^18^, ^20^, ^21^, ^22^, ^23^, ^26^, ^27^, ^28^ and 9 had a combination of surgical procedures performed.^9^, ^11^, ^13^, ^14^, ^15^, ^17^, ^19^, ^24^, ^25^ For the anesthetic, 10 patients had general anesthesia.^9^, ^10^, ^11^, ^13^, ^14^, ^17^, ^19^, ^21^, ^22^, ^25^ 7 patients had epidural anesthesia,^12^, ^13^, ^16^, ^17^, ^18^, ^20^, ^27^ 1 patient had spinal anesthesia,^23^ 7 had combined epidural and general anesthesia,^8^, ^9^, ^10^, ^15^, ^19^, ^24^, ^26^ 1 had general anesthesia with lumbar drain,^28^ and 1 had no anesthetic for labor.^1^ The results are summarized in Table 1.

**TABLE 1:** Case reports on recommendations regarding neuraxial anesthetic techniques in von Hippel‐Lindau disease

Author, Year	Age, sex	Presentation	Neuraxial imaging studies	Surgical procedure	Anesthetic	Outcomes	Recommendation/opinion
Matthews et al., 1986^12^	21, F	G1P0 at 39 weeks gestation	No	C‐section	Epidural anesthesia (L2‐3)	Uncomplicated postoperative course	In the absence of contraindication, lumbar epidural anesthesia can be considered for obstetric cases
Joffe et al., 1993^11^	35, F	G2P0 for antenatal assessment at 22 weeks gestation with new diagnosis of pheochromocytoma	No	C‐section and phaeochromocytoma resection	GA	Discharged a week after surgery	Neuraxial anesthesia is contraindicated due to potential HB rupture in presence of unknown intracranial and spinal lesions in VHLD
Ogasawara et al., 1995^13^	23, F	G3P0 at 35 weeks gestation with acute lower body sensory and motor loss, and urinary retention	MRI—Intermedullary hemorrhage at T4‐5 and intact HB at T7‐8 on admission	Emergency decompression and laminectomy at T3‐6 C‐section at 37 weeks gestation	GA Epidural anesthesia (T4‐5)	Uncomplicated postoperative course	Neuraxial imaging studies are recommended to identify HBs and CNS abnormalities to guide treatment
Mugawar et al., 1998^15^	22, M	Pheochromocytoma	Head CT—Large right cerebellar cystic lesion, two small left and right cerebellar lesions, and dilated ventricles	Emergent occipital craniectomy Adrenalectomy two weeks later	GA with epidural anesthesia	Discharged on steroid replacement therapy	No specific recommendations were discussed
Wang et al., 1999^16^	45, F	G2P1 at 38 weeks gestation for C‐section	Recent spine MRI—Small dorsal HB at T8‐9 and L2 with no cord compression	C‐section	Epidural anesthesia (L3‐4)	No neurological deficits two months post‐partum	Anesthesia technique should be tailored to the individual case including review of imaging studies. Epidural anesthesia should not be excluded based on VHLD diagnosis
Delisle et al., 2000^17^	35, F	Multigravida at 30 weeks gestation for headaches, diplopia, and unsteady gait	CT and MRI—Cerebellar cystic lesion and obstructive hydrocephalus. MRI at 38 weeks gestation with no spinal HB	Suboccipital craniotomy at 30 weeks gestation Forceps‐assisted vaginal delivery at 41 weeks gestation	GA Epidural anesthesia	Successful delivery	Anesthesia technique should be tailored to the individual case. Difficult to make recommendations, but epidural anesthesia is reasonable provided there are no contraindications. MDT approach for pregnant patients with VHLD
Boker et al., 2001^14^	30, F	G1P0 at 35 weeks gestation for anesthetic assessment and headaches	Brain and spine MRI at 36 weeks—Enlarging left cerebellar tumor	C‐section and posterior fossa craniotomy	GA	Uncomplicated postoperative course	Asymptomatic lesions may cause complications and comprehensive anesthetic assessment is mandatory
Demiraran et al., 2001^18^	23, F	G1P0 at 38 weeks gestation for C‐section	MRI—HB in bilateral retinas and cerebellum, and a renal cyst	C‐section	Epidural anesthesia (L3‐4)	Uncomplicated postoperative course/ Cerebellar tumor resection 2 months post‐partum	Anesthesia technique should be tailored to the individual case including review of imaging studies. Epidural anesthesia should not be excluded based on VHLD diagnosis
Gurunathan et al., 2004^9^	13, F	Intracranial hypertension and occult pheochromocytoma	MRI on admission—Cystic mass lesion in vallecula extending to vermis and inferior fourth ventricle	Suboccipital craniectomy for excision of hemangioblastoma and C1 arch Adrenalectomy three weeks later	GA GA and epidural anesthesia (T12‐L1)	Discharged	Full body imaging studies to detect other features to VHLD
Goel et al., 2005^19^	36, M	Intracranial hypertension and pheochromocytoma	Brain MRI—right cerebellar hemangioblastoma and obstructive hydrocephalus	Emergent craniotomy Bilateral adrenalectomy 10 days later	GA GA and epidural anesthesia	Good outcome/ Neurological outcome not reported.	No specific recommendations were discussed
Dubey et al., 2005^26^	26, F	G3P0 with pheochromocytoma	MRI not performed due to economic reason	Bilateral adrenalectomy	Epidural and general anesthesia (T11‐12)	Discharged/ Epidural analgesia for labor at a different facility	MRI or CT should be obtained to exclude HBs. Epidural anesthesia thought to be safe due to the natural distribution of HBs if dural puncture is avoided
Murthy et al., 2006^21^	21, M	Right lower limb weakness, backache, hypertension, and retinal angiomas	MRI on admission—Multiple cerebral hemangioblastomas and syrinx, spinal HBs, and bilateral renal cysts	Occipital craniotomy and spinal cyst excision	GA	Uneventful postoperative course and discharged/ No neurological sequala	Authors preferred avoiding epidural analgesia in the presence of spinal HBs
Junglee et al., 2007^20^	22, F	G3P2 at 39 weeks gestation with pheochromocytoma	Brain and spine MRI—Normal study	Spontaneous vaginal delivery with vacuum‐assisted delivery	Epidural analgesia	Uneventful postoperative course/ Bilateral adrenalectomy 6 weeks post‐partum	Anesthesia technique should be tailored to the individual case with MDT approach for optimal outcome
Razvi et al., 2009^22^	30, F	G2P0 for antenatal assessment at 37 weeks gestation/ Lumbar puncture at 10 weeks gestation for headaches	Brain CT at 10 weeks gestation—stable temporoparietal and cervical HBs.	C‐section	GA	Uneventful postoperative course	Anesthesia technique should be tailored to the individual case including taking into account patient's wishes, MDT discussion, and updated neuraxial imaging especially if neuraxial anesthesia is being contemplated
McCarthy et al., 2010^23^	26, F	G6P5 at 36 weeks gestation for urgent C‐section	MRI at 36 weeks gestation—No cerebellar lesion and stable spinal lesions, small anterior T9 and T10 posterolateral lesion	C‐section	Spinal anesthesia (L3‐4)	No neurological deficit post‐partum	No specific recommendations were discussed, but authors describe excluding contraindications to spinal anesthesia such as raised ICP, and absence of space‐occupying lesion
Adekola et al., 2013^1^	26, F	G1P0 at 18 weeks gestation for prenatal care	MRI during pregnancy and 11 months prior—Intramedullary masses at C4, C6, T1/ Diffuse cord enlargement and edema from cervicomedullary region to T1.	Spontaneous vaginal delivery at 37 weeks and 3 days	Epidural analgesia planned based on MRI/ No epidural due to expeditious labor	Uneventful post‐partum period	Mode of delivery and anesthesia should be tailored to the individual case. Acknowledges there are no recommendations for obstetrical anesthesia, and there have been no reported complications with neuraxial anesthesia in patients with VHLD
Lam et al., 2014^10^	9, M	Pheochromocytoma	Brain MRI on admission—ischemic stroke in brain	Bilateral pheochromocytoma resection Excision of recurrent left adrenal tumor a year later	GA GA and epidural anesthesia	Left leg numbness seven months postoperatively No neurological deficits	No specific recommendations were discussed
Mungasuvalli et al., 2014^24^	24, M	Pheochromocytoma	Brain and spine CT and MRI on admission—Cerebellar hemisphere, medullary and C7 HBs, dilation of 3rd and lateral ventricles, compression of fourth 4th, and syrinx from C2‐T10	Emergent VP shunt Laparoscopic adrenalectomies	Not specified GA and epidural anesthesia (T12‐L1)	Uneventful postoperative course	No specific recommendations were discussed
Hallsworth et al., 2015^25^	37, F	G2P1 at 26 weeks gestation/ Symptoms of elevated ICP	MRIx2 during pregnancy—Edematous cerebellar tumors/ Known T3 and L1 HB	ICP monitor placement and C‐section	GA	Neurologically intact after extubation/ Intracranial tumor excision seven months post‐partum	Neuraxial anesthesia can be considered but neuroimaging must be obtained. Neuraxial anesthesia is an absolute contraindication if HB lesions are close to puncture site
Dias et al., 2015^8^	11, M	Pheochromocytoma	No	Bilateral adrenalectomies and Whipple's procedure	GA and epidural anesthesia (T9‐10)	Discharged	No specific recommendations were discussed
Lenk et al., 2016^27^	33, F	G2P1 at 34 weeks gestation with neck stiffness and bilateral shoulder pain	MRI—Cervical cord edema and no lumbar HB lower than L2	Spontaneous labor	Epidural analgesia (L3‐4)	No complications following epidural removal	Epidural anesthesia is appropriate if imaging studies demonstrate no HBs and no raised ICP. Neuraxial anesthesia is contraindicated in the presence of HB. MDT approach is essential

Abbreviations: C, cervical vertebrae; CNS, central nervous system; C‐section, Cesarean section; CT, computed tomography; F, female; G, gravida; P para; GA, general anesthesia; HB, hemangioblastoma; ICP, intracranial pressure; ICP, intracranial pressure; L, lumber vertebrae; M, male; MDT, multidisciplinary team; MDT, multidisciplinary team; MRI, magnetic resonance imaging; T, thoracic vertebrae; VHLD, von Hippel‐Lindau disease; VP, ventriculoperitoneal.

**FIGURE 2:**
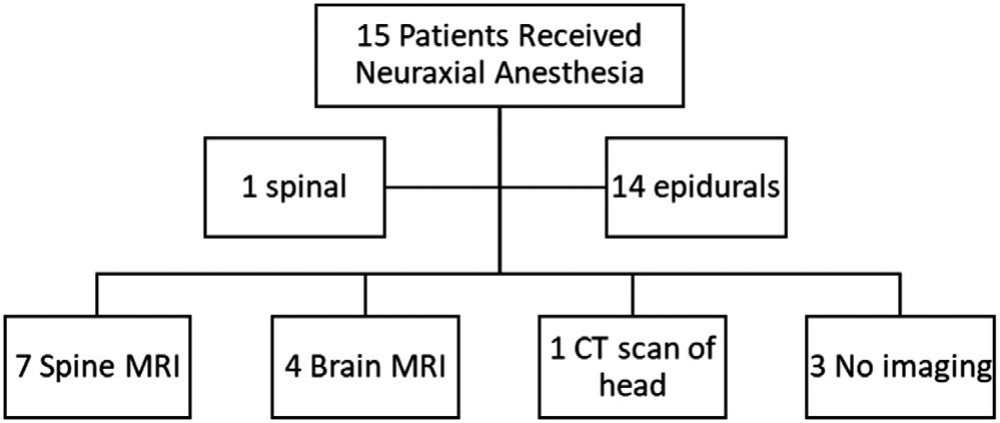
Neuraxial anesthesia and Imaging. MRI, magnetic resonance imaging; CT, computed tomography

Did practitioners obtain pre‐procedural neuraxial imaging study(ies)? Of the 15 patients who received neuraxial anesthesia, 14 had epidurals^8^, ^9^, ^10^, ^12^, ^13^, ^15^, ^16^, ^17^, ^18^, ^19^, ^20^, ^24^, ^26^, ^27^ while 1 patient had spinal anesthetic for a cesarean section.^23^ Of these 15 patients, 7 had an MRI of the spine reported,^13^, ^16^, ^17^, ^20^, ^23^, ^24^, ^27^ while 5 had MRI/CT of the brain^9^, ^10^, ^15^, ^18^, ^19^ with no details of spine imaging reported in the article. There were 3 patients without any imaging studies who received neuraxial anesthesia.^8^, ^12^, ^26^ Thus, of the 15 patients who had a neuraxial anesthesia, only 7 (47%) had pre‐procedural imaging of the spine (Figure 2). Expert opinion presented at the 2013 European Society of Regional Anesthesia Congress recommends that patients with VHLD should have an MRI performed as closely to the planned neuraxial anesthetic technique as possible and that neuraxial anesthesia should be avoided if imaging is unavailable.^29^ Nevertheless, fewer than half of the patients (7/15) included in this literature review had imaging studies of the spinal cord prior to administration of neuraxial anesthesia.

What recommendations had been made regarding performing neuraxial anesthesia techniques in patients with VHLD? 5 groups did not provide any opinion or recommendations with regard to neuraxial anesthesia techniques in patients with VHLD.^8^, ^10^, ^19^, ^24^, ^28^ 10 groups suggested that neuraxial anesthesia can be considered for use in VHLD patients in the absence of contraindications.^1^, ^12^, ^16^, ^17^, ^18^, ^20^, ^22^, ^25^, ^26^, ^27^ 9 groups advised reviewing neurological imaging prior to performing neuraxial anesthesia techniques^9^, ^13^, ^16^, ^18^, ^20^, ^22^, ^25^, ^26^, ^27^ with 1 group specifically stating that updated neurological imaging should be acquired.^22^ 1 group stated that neuraxial anesthesia is an absolute contraindication in VHLD patients due to the potential presence of spinal hemangioblastomas^11^ but most others stated that the diagnosis of VHLD should not completely exclude the use of neuraxial anesthesia.

What were the outcomes in patients with VHLD who received neuraxial anesthesia techniques? We found no reports of complications following neuraxial anesthesia techniques in the 15 VHLD patients included in this review.

## 4 CONCLUSION

Spinal hemangioblastomas in patients with VHLD may be ruptured by neuraxial instrumentation. However, in the absence of spinal hemangioblastoma close to the site of needle entry, neuraxial anesthesia can be used safely. Nevertheless, there are no specific guidelines for neuraxial anesthesia, and recommendations and opinions differ among the reported literatures. The current evidence is insufficient to determine if neuraxial anesthesia is safe or contraindicated in VHLD in the absence of spine imaging. Therefore, the decision should be made on a case‐by‐case basis with the risks and benefits in mind.

## CONFLICT OF INTEREST

The authors have no conflicts of interest to declare.

## AUTHOR CONTRIBUTIONS

AY and YSR performed the literature search. All authors contributed to the study conception and design, drafted and/or critically revised the work, read and approved the final manuscript.

## CONSENT

Written informed consent was obtained from the patient for the publication of this case report.

## Data Availability

Data sharing is not applicable to this article as no new data were created or analyzed in this study.
